# Pustular Eruption of the Skin from the Internal Use of Tartar Emetic

**Published:** 1856-02

**Authors:** 


*Pustular Eruption of the Skin from the Internal use of Tartar Emetic.*

Dr. BOISLINIERE relates in the St. Louis *Med. and Surg. Journal,* a few cases in which emetic tartar administered internally in cases of pneumonia, produced its characteristic pustules upon the skin. He infers from this that when used for pneumonia its beneficial effects are due to its absorption and elective or *specific* action on the mucous membrane of the lungs, which *in certain cases* extends itself to the skin, by virtue of the continuity of tissue between it and the mucous membrane of the lungs.

